# Cardio-Renal Metabolic Syndrome: Interdisciplinary Diagnostic Methods

**DOI:** 10.3390/diagnostics13020265

**Published:** 2023-01-11

**Authors:** Simona Gabriela Bungau

**Affiliations:** 1 Department of Pharmacy, Faculty of Medicine and Pharmacy, University of Oradea, 410028 Oradea, Romania; sbungau@uoradea.ro; 2 Doctoral School of Biomedical Sciences, University of Oradea, 410087 Oradea, Romania

Medical interdisciplinarity in making a correct diagnosis is of the utmost importance for an optimal treatment, which should include both effective therapeutic means (drugs and/or surgery) and the complex aspects (nutrition, lifestyle, rehabilitation, etc.).

This Special Issue intends to explore scientific and experimental data and information relating to modern and complex interdisciplinary diagnosis, in directions such as:

- New methods for evaluating metabolic cardio-renal diseases;
- Nutritional, pharmacological, and rehabilitation interventions in metabolic diseases and their complications;
- Diagnostic algorithms from medical history/clinical picture to imaging, biochemical, molecular, and genetic tests;
- New markers for assessing the cardio-metabolic risk, such as clinically significant molecules, intestinal microbiota, etc.;
- The role of plant-derived natural compounds/biologically active phytochemicals in chronic metabolic diseases;
- The impact of novel guidelines in real-life studies for the diagnosis and treatment of cardio-metabolic diseases;
- Treatments with beneficial effects in the risk of cardiovascular morbidity and mortality;
- Interdisciplinarity in diagnoses and interventions.

In the respect to the above-mentioned data, Gheorghe et al. reviewed the cardiovascular risk and statin therapy considerations to reveal particularities relating to dyslipidemia and lipid-lowering therapies in women [1]. Moreover, Gheorghe et al. provided a complex and very useful framework regarding the early diagnosis of pancreatic cancer (PC), which is considered by the authors to be key for survival; the paper also presents the most important diagnostic tools (specific risk factors, symptoms of the disease, laboratory tests, investigation methods, etc.), which are at hand for an early diagnosis of PC [2].

Another interesting review was provided by Peterson et al., which focused on evaluating the role of high-density lipoprotein (HDL) and the pathway of the reverse cholesterol transport in cardiovascular–renal pathophysiology. The results of their literature study concluded that heme-oxygenase-1 (HO-1) can be suggested as a new therapeutic target to prevent HDL dysfunction, as well as to implicitly prevent cardiovascular/ renal dysfunction and the occurrence of cardio-renal syndrome [3].

Moreover, through a review, Pafundi et al. documented and updated the information regarding the importance of albuminuria in diabetic patients in the context of cardio-renal risk [4].

Another review performed by Jichitu et al. addresses the pathogenic mechanisms that induce two extremely frequent diseases (namely the non-alcoholic fatty liver disease known as NAFLD, as well as cardiovascular disease), while presenting the current state of the modern therapeutic management [5]. These authors concluded that a multidisciplinary approach is needed to control the risk factors and to prevent the appearance of cardiovascular and liver complications. 

Moisi’s team also carried out a study of the literature, with a specific focus on acute coronary syndrome in subjects with chronic kidney disease, presenting the newest perspectives vis-a-vis the cause–effect relationship of the association of the two diseases [6].

No less important is the article by Hernández-Reyes et al., which carries out a cross-sectional pilot study among young adult men, and uses waist circumference (instead of body mass index) as an important preventive tool in the case of atherogenic dyslipidaemia and obesity-associated cardiovascular risk [7].
